# Microstructure and Mechanical Properties of Carboxylated Nitrile Butadiene Rubber/Epoxy/XNBR-grafted Halloysite Nanotubes Nanocomposites

**DOI:** 10.3390/polym12051192

**Published:** 2020-05-23

**Authors:** Seyed Mohamad Reza Paran, Ghasem Naderi, Heydar Mosallanezhad, Elnaz Movahedifar, Krzysztof Formela, Mohammad Reza Saeb

**Affiliations:** 1 Department of Polymer Processing, Iran Polymer and Petrochemical Institute, P.O. Box 14965/115, Tehran, Iran; g.naderi@ippi.ac.ir (G.N.), ahmn3021@gmail.com (H.M.); el.movahedifar@gmail.com (E.M.); 2 Department of Polymer Technology, Faculty of Chemistry, Gdańsk University of Technology, Gabriela Narutowicza 11/12, 80–233 Gdańsk, Poland; 3 Departments of Resin and Additives, Institute for Color Science and Technology, P.O. Box 16765-654, Tehran, Iran; mrsaeb2008@gmail.com

**Keywords:** carboxylated nitrile butadiene rubber, epoxy, halloysite nanotubes, mechanical behavior, Bergström–Boyce model, swelling

## Abstract

The effect of various amounts of carboxylated nitrile butadiene rubber (XNBR) functionalized halloysite nanotubes (XHNTs) on the cure characteristics, mechanical and swelling behavior of XNBR/epoxy compounds was experimentally and theoretically investigated. The morphology of the prepared XNBR/epoxy/XHNTs nanocomposites was imaged using scanning electron microscopy (SEM). The effects of various XNBR-grafted nanotubes on the damping factor of nanocomposites were evaluated by dynamic mechanical thermal analysis (DMTA). The cure behavior characterization indicated a fall in the scorch time, but a rise in the cure rate with higher loading of XHNTs into the XNBR/epoxy nanocomposites. SEM micrographs of tensile fracture surfaces were indicative of a rougher fracture surface with a uniform dispersion state of nanotubes into the polymer matrix in the XNBR/epoxy/XHNTs nanocomposites. The stress–strain behavior studies of XNBR/epoxy/XHNTs nanocomposites showed a higher tensile strength up to 40% with 7 wt % XHNTs loading. The theoretical predictions of uniaxial tensile behavior of nanocomposites using Bergström–Boyce model revealed that some of the material parameters were considerably changed with the XHNTs loading. Furthermore, the used theoretical model precisely predicted the nonlinear large strain hyperelastic behavior of nanocomposites.

## 1. Introduction

Rubber composites and nanocomposites have received continued attention in recent years [1,2,3]. A wide variety of nanoparticles were examined in detail in various rubber matrices to achieve higher properties [4,5,6]. Carboxylated nitrile butadiene rubber (XNBR) is a special type of nitrile butadiene rubber (NBR) containing carboxyl functional groups which cause to exhibit enhanced tear and abrasion resistance [7]. Whereas the oil and solvent resistance properties of the rubber will retain at the excellent level [8]. The vulcanization of XNBR can form different types of chemical bonds due to the presence of variety of functional groups in the polymer chain backbone containing nitrile, carboxylic and alkene groups [9]. The carboxylic functional group in the chemical structure of XNBR can react with several materials such as metal oxides, amines, polyols and epoxies in the curing process [10].

The carboxylic functional group creates a potential for XNBR to mix with various fillers and polymers with sufficient interfacial interactions in the mixing and curing processes such as epoxy polymers. Epoxy resins are widely used in various applications such as thermosetting composites [11], thermal conductive nanocomposites [12] and honeycomb sandwich panels for aerospace applications [13]. Chakraborty et al. [9] studied the properties of XNBR/epoxy blends in presence of carbon black and found that 7.5 parts per hundred rubber (phr) of epoxy resin leads to a compound with optimum cure behavior and mechanical properties. Laskowska et al. [14] investigated the effect of various magnesium aluminum layered double hydroxide (MgAl–LDH) on the properties of XNBR and represented that the incorporation of LDH into the XNBR matrix had a significant impact on the glass transition temperature (T_g_), cure behavior and mechanical properties. Sahoo et al. [15] studied the effect of introduction of nano zinc oxide (ZnO) on the cure characteristics and mechanical properties of XNBR and confirmed a better state of cure and higher mechanical propertied in comparison with the conventional ZnO.

Halloysite nanotubes (HNTs) are naturally occurs nano cylinders which recently used in various polymer nanocomposites for their high thermal and mechanical properties [16,17,18]. We have experimentally studied the effect of HNTs on the physical and mechanical properties of various polymer matrices. The nanocomposites of polyamide 6 (PA6)/nitrile butadiene rubber (NBR) thermoplastic elastomers (TPEs) containing various concentrations of pristine and silane modified HNTs were investigated and found that the introduction of silane modified HNTs into the PA6/NBR TPEs cause a rise in the tensile strength and Young’s modulus of polymer matrix due to the physical structure of the nanotubes and their interactions with PA6 [19]. Our following researches focused on the effect of HNTs on the crystallization [20] and degradation [21] behavior of dynamically vulcanized PA6/NBR thermoplastic elastomer vulcanizates (TPVs). The results indicated a higher thermal stability for nanocomposites containing higher HNTs loading. In our previous investigations [22], we stated that the surface modified HNTs with silane functional groups could be grafted with XNBR. The resulted XNBR grafted HNTs has a great potential as a reinforcing agent in many polymer systems.

Our findings suggested a detailed study of the cure behavior and mechanical characteristics of the XNBR/epoxy nanocomposites containing various concentrations of XNBR grafted halloysite nanotubes (XHNTs). The main objective of this research focused on the theoretical and experimental evaluations of the stiffness and stress–strain behavior of the XNBR/epoxy/XHNTs nanocomposites through using appropriate large strain hyperelastic theoretical models. The predictions on the stiffness analysis and uniaxial stress–strain behavior of nanocomposites were evaluated in comparison with the experimental results of tensile experiments of XNBR/epoxy/XHNTs containing various nanotube loadings.

## 2. Theoretical Background

The mechanical behavior of large strain rubber like materials such as XNBR/epoxy/XHNTs nanocomposites could be predicted through using Bergström–Boyce model which was discussed in details in our previous work [23]. The uniaxial stress–strain behavior of a polymer with large strain deformation could be represented in two parallel networks with a hyperelastic behavior and time dependent viscoelastic response [24]. The Cauchy stress tensor for hyperelastic response of a rubber like material is given by the following equation [25]:

(1) $$T_{A}=\frac{\mu }{J{\bar{\lambda }}^{*}}\frac{L^{-1}({\bar{\lambda }}^{*}/\lambda_{L})}{L^{-1}(1/\lambda_{L})}dev[B^{*}]+\kappa [\ln J]I$$

In contrast, the Cauchy stress tensor for time dependent viscoelastic behavior could be represented as [25]:

(2) $$T_{B}=\frac{s\mu }{J_{B}^{e}{\bar{\lambda }}_{B}^{e*}}\frac{L^{-1}({\bar{\lambda }}_{B}^{e*}/\lambda_{L})}{L^{-1}(1/\lambda_{L})}dev[B_{B}^{e*}]+\kappa [\ln J_{B}^{e}]I$$

where, μ and κ are the shear and bulk moduli, $\lambda_{L}$ is defined as the limiting chain stretch, I is the second-order identity tensor and $L^{-1}(x)$ represented as the inverse Langevin function. The parameter J is the Jacobian and ${\bar{\lambda }}^{*}$ is the applied chain stretch. The ratio of shear modulus of viscoelastic response to the shear modulus of hyperelastic response of material represented as *s* in Equation (2) which is a dimensionless material parameter.

There are some of the material parameters in the Bergström–Boyce model which could be affected by incorporation of XHNTs into the XNBR/epoxy matrix. These material parameters could be separately calculated for each nanocomposite by matching the experimental data of tensile tests with the theoretical model through using an optimization method.

## 3. Experimental

### 3.1. Materials

Carboxylated nitrile butadiene rubber (XNBR) was Krynac X160, supplied by Lanxess Elastomers (Dormagen, Germany) which contains 32.5% by weight of acrylonitrile and 1% by weight of carboxylic acid group. The diglycidyl ether of bisphenol A (DGEBA) type epoxy resin, KER828, with epoxy group content of 5260–5420 mmol/kg, was obtained from Kumho P&B Chemicals, Seoul, South Korea. XNBR grafted halloysite nanotubes (XHNTs) synthesized in accordance with our previous works through using halloysite nanotubes (HNTs), ultrafine grade, was procured from Imerys Tableware Asia Limited (North Island, New Zealand). Other ingredients such as zinc oxide and acetic acid were laboratory reagent grades which prepared from Merck Co. (Darmstadt, Germany) and used as received.

### 3.2. Nanocomposite Preparation

**Scheme 1:**
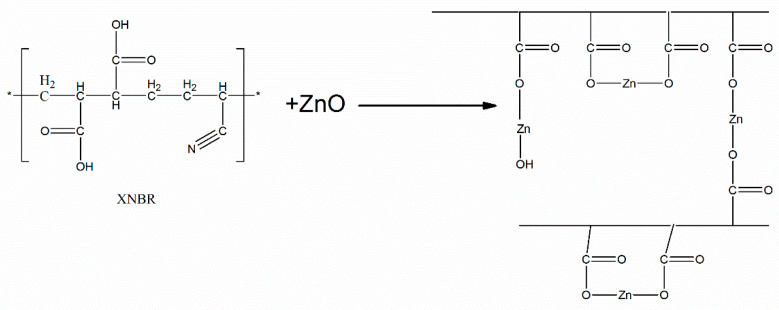
Possible reactions between ZnO and carboxylic groups in XNBR.

The XNBR/epoxy/XHNTs nanocomposites with various formulations in accordance with Table 1, were prepared on a laboratory open two roll mills, running at speed ratio of 1:1.2 for 10 min at 40 °C. At first stage, The XNBR was masticated for 1 min and then the epoxy resin was added to the rubber. The XHNTs was incorporated into the rubber mixture after 2 min of mixing process and the mixing was continued for 5 min. Finally, the ZnO and acid stearic were added to the nanocomposite as a curing agent and activator which mixed to the rubber for 3 min. As indicated in Scheme 1, the ZnO can react with carboxylic group of XNBR and acts as a curing agent for this rubber. The prepared rubber compounds were compression molded in a compression molding machine at 175 °C and time needed to reach the required optimum cure according to the optimum cure time obtained from Monsanto Oscillating Disc Rheometer R-100 (Monsanto Company, St. Louis, MO, USA).

### 3.3. Characterization

The morphology of XNBR/epoxy/XHNTs nanocomposites was determined by Vega II XMU scanning electron microscope (SEM) from Tescan Brno s.r.o., Brno, Czech Republic. The SEM analysis was carried out on the cryogenically fractured surfaces after coating with gold powders by sputtering technique.

The cure behavior of prepared samples was studied by using a Monsanto Rheometer R-100 testing instrument operated at 175 °C with 3° arc at a period of 30 min in accordance with ASTM D2084.

The uniaxial stress–strain behavior of XNBR/epoxy nanocomposites containing various XHNTs loading was monitored according to ASTM D412 through using Universal tensile testing machine, Instron 6025 model (Instron Ltd., Norwood, MA, USA) operated at room temperature at an extension speed of 500 mm/min with an initial gauge length of 25 mm.

The phase structure of the prepared nanocomposites was evaluated from dynamic mechanical thermal analysis (DMTA) using Triton Technology Tritec 2000DMA (Nottinghamshire, UK). The storage modulus and damping factor of prepared samples were recognized in tension mode at a constant heating rate of 3 °C/min and a frequency of 1 Hz in a strain of 0.02 mm from −100 °C to 100 °C.

Swelling of rubber nanocomposites was extensively studied [26,27] Swelling of the various XNBR/epoxy/XHNTs nanocomposites was investigated in toluene solvent in accordance with ASTM D5964. The needed samples for swelling test were cut from the molded slabs and weighted in dry state. The swollen weights of the samples immersed in the solvent for 72 h were recorded to determine the swelling ratio and cross-link density through using Flory–Rehner equations [28]:

(3) $$Q_{s}=\frac{w_{s}-w_{u}}{w_{u}}$$

(4) $$\upsilon_{sw}=\frac{-[\ln(1-\upsilon )+\upsilon +\chi \upsilon^{2}]}{V_{s}(\upsilon^{\frac{1}{3}}-\frac{\upsilon }{2})}$$

where $Q_{s}$ defined as the swelling ratio, $w_{s}$ and $w_{u}$ are the swollen and unswollen weights of the sample, respectively. The parameter, $\upsilon_{sw}$, is the cross-link density (mol/m^3^), χ is the polymer-solvent interaction parameter, $V_{s}$ is molar volume of the solvent (m^3^/mol) and υ is the volume fraction of polymer in swollen state which could be calculated from the following equation [29]:

(5) $$\upsilon =\frac{\frac{w_{p}}{d_{p}}}{\frac{w_{p}}{d_{p}}+\frac{w_{s}}{d_{p}}}$$

where $w_{p}$ and $w_{s}$ are the weight fractions of rubber and solvent in the swollen sample, respectively. The parameters $d_{p}$ and $d_{s}$ are defined as the densities of polymer and solvent, respectively. The polymer-solvent interaction parameter could be calculated using following equation [30]:

(6) χ=0.487+0.228υ

Another approach to determine the cross-link density of a cured rubber system is the following equation which uses a hypothesis that the cross-link density has a direct relation with the Young’s modulus of the rubber [31]:

(7) $$\upsilon_{e}=\frac{E}{3RT}$$

where E is the Young’s modulus which determined from the slope of stress–strain cure at the initial region of elongation, R is universal gas constant (8.314 J/mol·K) and T is the absolute temperature (K).

Furthermore, the cross-link density parameter of a cured rubber could be calculated using the modulus at the rubbery plateau region in the plot of storage modulus versus temperature [31]:

(8) $$\upsilon_{st}=\frac{E_{st}}{6RT}$$

where $E_{st}$ is the storage modulus at the rubbery plateau region.

## 4. Results and Discussion

### 4.1. Cure Characteristics

**Figure 1:**
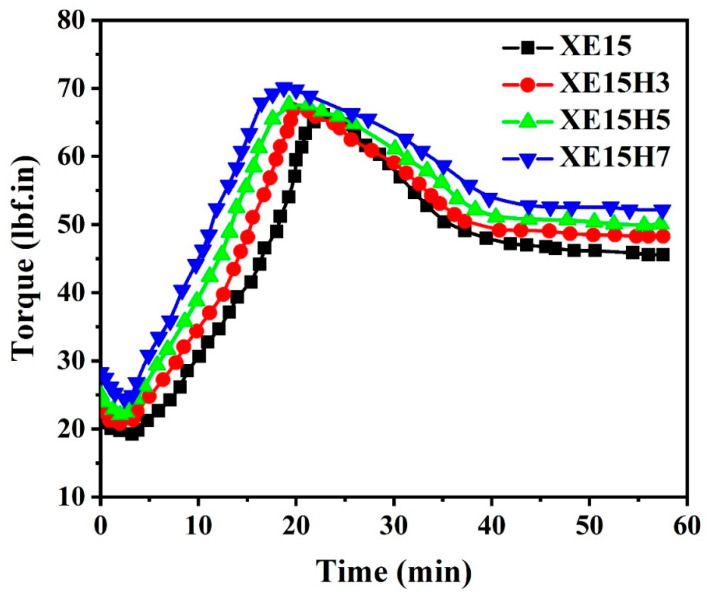
Cure behavior of various XNBR/epoxy/XHNT s nanocomposites.

The cure behavior of the XNBR/epoxy nanocomposites containing various XHNTs loading represented in Figure 1. It is obvious that the higher concentrations of XHNTs leads to a higher torque values at the initial and final stages of curing of nanocomposites. The relevant cure parameters for XNBR/epoxy compounds and its nanocomposites extracted from Figure 1, are summarized in Table 2. The results indicated that the scorch time and optimum cure time reduced with the introduction of XNHTs into the rubber formulation. It may be due to the interactions between the XHNTs and XNBR matrix which cause a rise in the torque rheometer and higher curing rate with respect to the neat XNBR/epoxy compound [32]. In addition, the minimum torque is much higher, and this parameter is related to the viscosity of the compound and it is very often in the XNBR [33]. The higher viscosity of these elastomers are related to the possibility of creating strong interactions between the carboxylic acids of these compounds [34]. In fact, the higher viscosity due to the strong interactions between carboxylic groups make difficult the mixing and processing of these compounds.

### 4.2. Morphologic Observations

**Figure 2:**
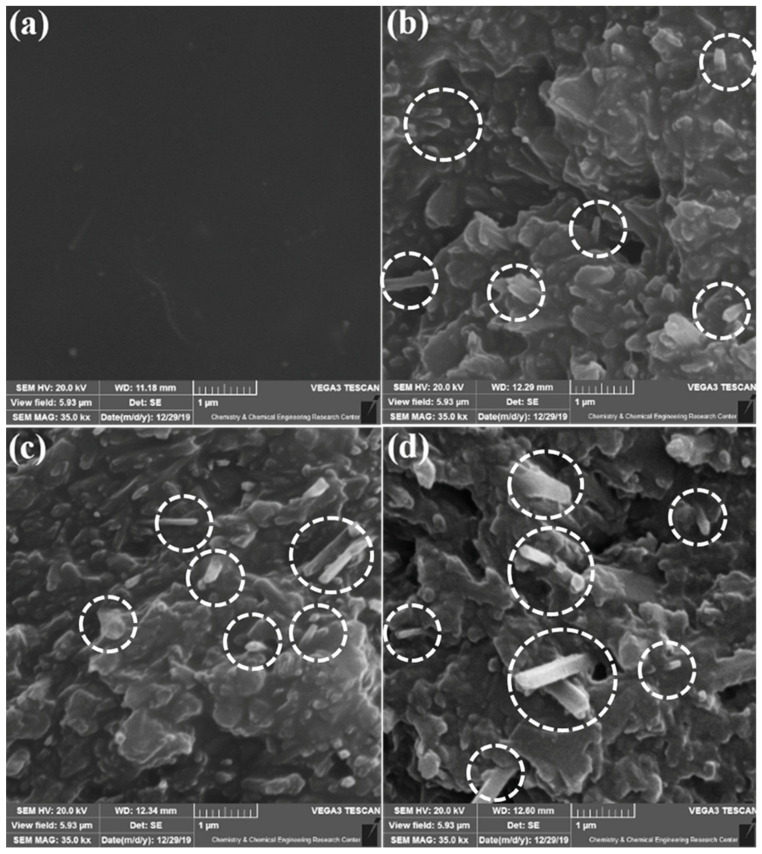
SEM photomicrographs of XNBR/epoxy nanocomposites containing various concentrations of XHNTs (**a**) 0 wt %, (**b**) 3 wt %, (**c**) 5 wt % and (**d**) 7 wt %.

The SEM photomicrographs of tensile fracture surfaced of XNBR/epoxy and XNBR/epoxy nanocomposite containing various loading of XHNTs are compared in Figure 2. As can be seen in Figure 2b–d the XNBR/epoxy/XHNTs nanocomposite has a rougher fracture surface in comparison with the neat XNBR/epoxy compound (Figure 2a) which can be resulted from the good dispersion of nanotubes into the polymer matrix [35]. Figure 2b–d show that the XNBR-grafted-HNTs (determined with dashed circles) has a uniform dispersion state in the XNBR matrix, so it is expected that the higher XHNTs loading leads to the higher mechanical behavior in the XNBR/epoxy compounds [36]. Furthermore, the SEM images show that the XHNTs have about 40 nm of average external diameter their average internal diameter is about 18 nm with a maximum length of 1 μm.

### 4.3. Mechanical Properties

**Figure 3:**
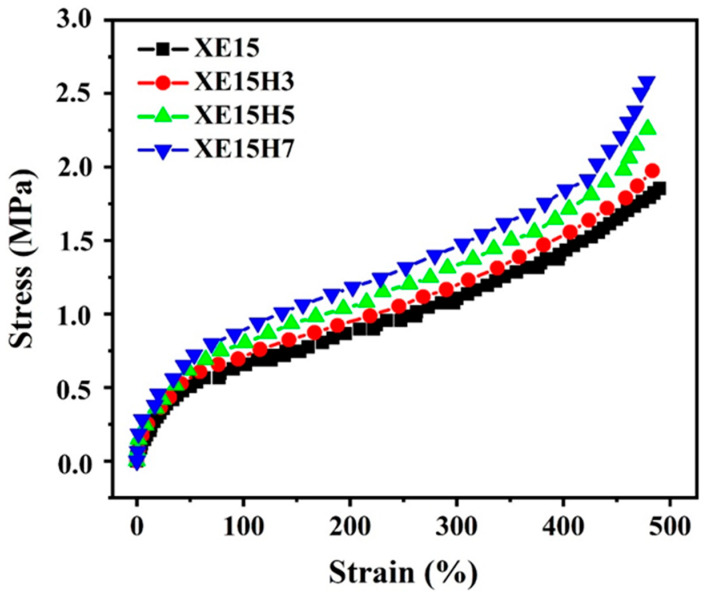
Uniaxial stress–strain behavior of XNBR/epoxy/XHNTs nanocomposites.

**Figure 4:**
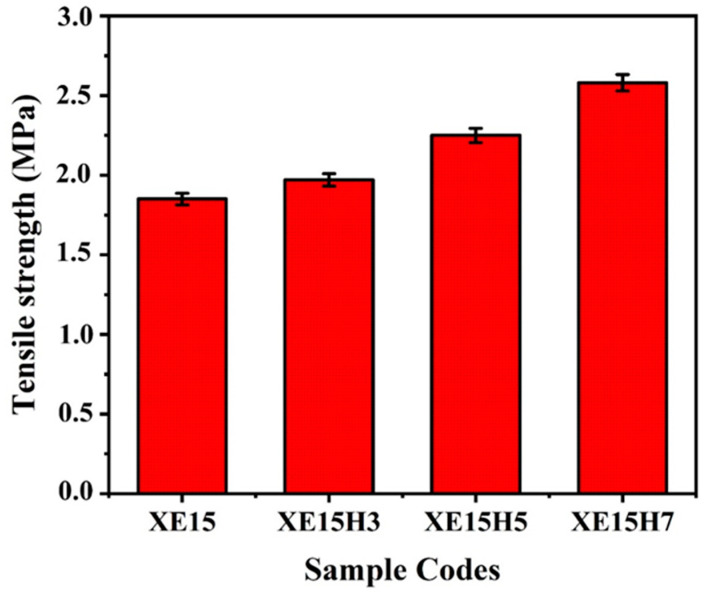
Tensile strength of various XNBR/epoxy/XHNTs nanocomposites.

**Figure 5:**
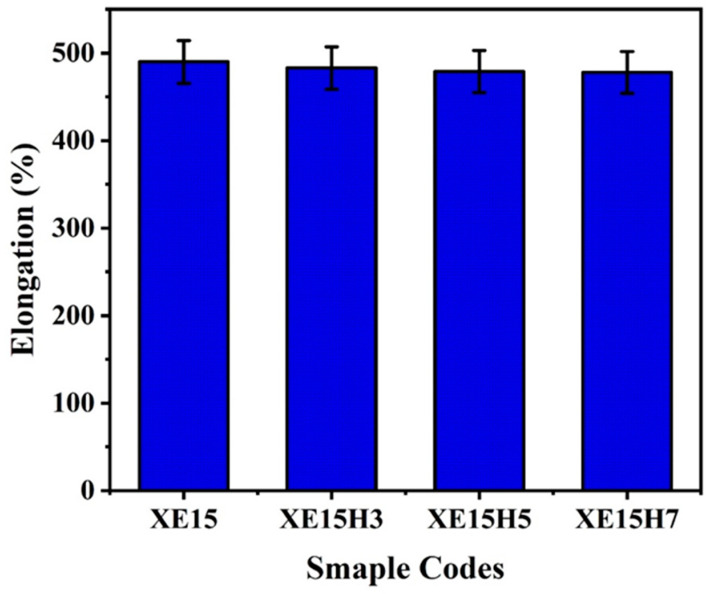
Elongation at break of various XNBR/epoxy/XHNTs nanocomposites.

**Figure 6:**
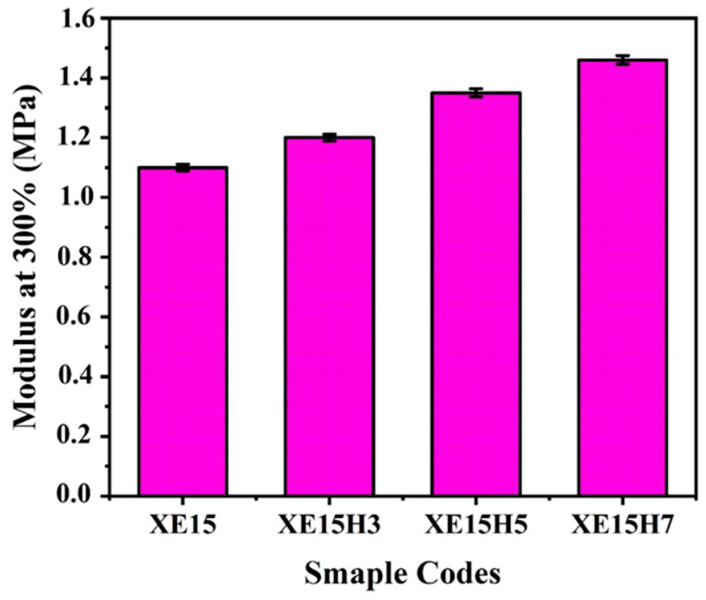
Young’s modulus at 300% elongation for various XNBR/epoxy/XHNTs nanocomposites.

The uniaxial stress-stain behavior of various XNBR/epoxy/XHNTs nanocomposites are depicted in Figure 3. We can see that, the introduction of XHNTs into the XNBR/epoxy compounds leads to a higher tensile behavior due to the stiffening effect of nanotubes and the interfacial interactions between the XHNTs and rubber matrix [37]. Figure 4 shows the effect of the nanotubes on the tensile strength behavior of nanocomposites, where a higher tensile strength up to 40% increase was achieved with the addition of XHNTs to the XNBR/epoxy compound. Furthermore, the nanocomposites containing 5 wt % of XHNTs show a higher increase in tensile strength compared to the XNBr/epoxy containing 3 wt % of nanotubes. However, there is some reduction in elongation at break with higher concentrations of nanotubes as depicted in Figure 5 due to some restrictions in chain mobility of polymer matrix induced by incorporation of nanotubes [38]. Figure 6 shows that the introduction of XHNTs into the XNBR/epoxy matrix leads to an increase in modulus at 300% elongation which is attributed to the dispersion state of nanotubes and their interactions with polymer matrix [39].

### 4.4. Stress–Strain Analyses

The uniaxial large strain behavior of various XNBR/epoxy/XHNTs was evaluated through using Bergström–Boyce model by the Nelder–Mead simplex optimization procedure [40] in MCalibration software. The effect of the various loadings of XHNTs on the model parameters was investigated and their variations were monitored in Table 3 which could be used to predict the hyperelastic behavior of nanocomposites. The results indicated the higher shear modulus, *μ,* with higher XHNTs concentrations which may be due to the induction of the chain mobility restrictions by nanotubes because of some interactions between the XHNTs and polymer matrix [41]. As indicated in Table 3, the bulk modulus, *κ*, is high for all samples due to the used incompressibility hypothesis in the modeling of stress–strain behavior [42]. Furthermore, it is evident from Table 3 that the bulk modulus increased with the higher concentrations of XHNTs due to the good dispersion of nanotubes and their interactions with the polymer matrix [43]. The results of the parametric study of Bergström–Boyce model in Table 3 show that the rate of stress relaxation, *s*, decreased with incorporation of nanotubes into the XNBR/epoxy matrix. Another parameter is the strain exponential factor, *C*, which increases with higher loadings of XHNTs. It should be noticed that when this parameter tends toward −1, the mechanical behavior of material become more hyperelastic [44]. Consequently, the results of parametric study of model show that the introduction of nanotubes decreases the elastomeric mechanical behavior of XNBR/epoxy/XHNTs. The flow resistance, $\tau_{base}$ variations show an increase with higher XHNTs loading due to the higher internal dynamic friction of polymer matrix induced by some interactions between the nanotubes and polymer chains [45]. Therefore, the results of static internal friction factor, *τ_cut_*, indicated the higher values with XHNTs concentrations.

**Figure 7:**
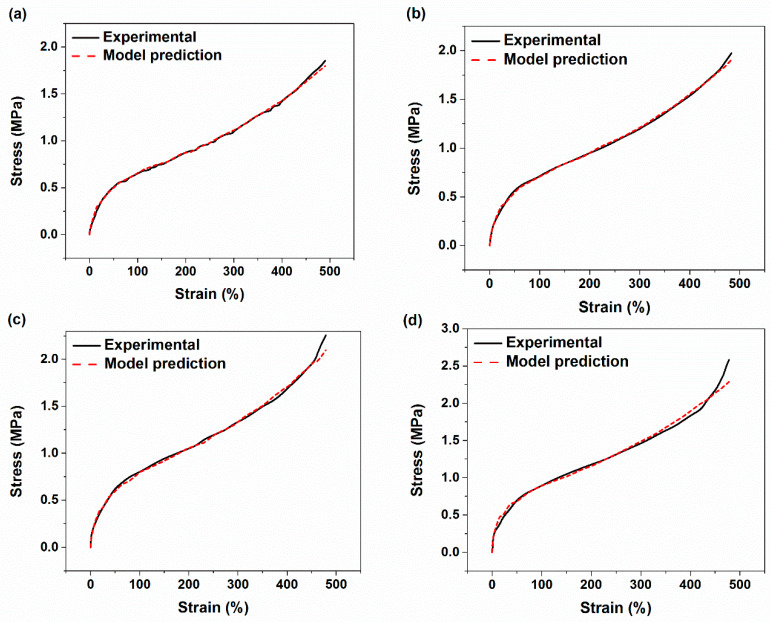
Comparison between the experimental values and model prediction on uniaxial tensile behavior for various XNBR/epoxy/XHNTs nanocomposites (**a**) XE15 (**b**) XE15H3 (**c**) XE15H5 (**d**) XE15H7.

A comparison between the experimental data of uniaxial stress–strain behavior of prepared nanocomposites and predicted results was carried out as shown in Figure 7. We can see that there is a good agreement between the experimental and model predicted results for all prepared samples. However, there is some deviations from experimental data especially at the region of ultimate elongations. Furthermore, the incorporation of higher values of XHNTs into the polymer matrix leads to more deviations from experimental uniaxial data. It may be due to some additional failure mechanism such as stretch debonding at the region of ultimate elongation induced by the nanotubes [46].

### 4.5. DMTA Investigations

**Figure 8:**
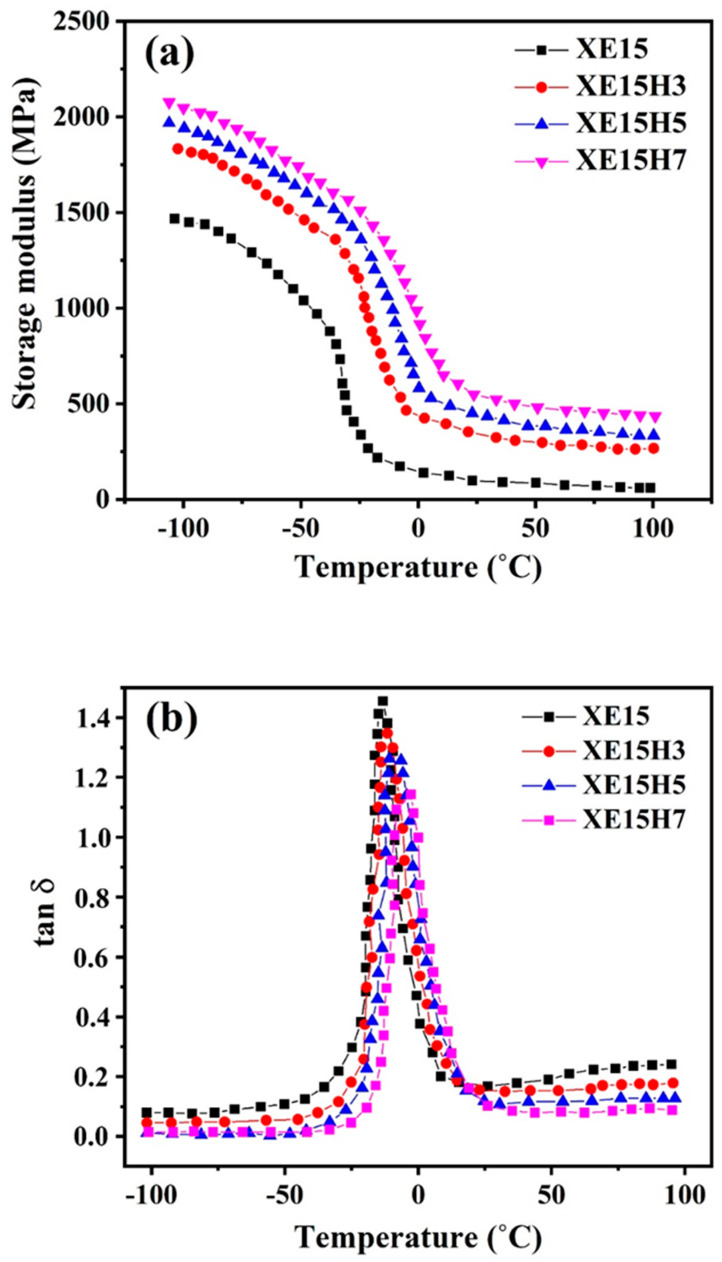
The variations of storage modulus (**a**) and tanδ (**b**) with temperature for various XNBR/epoxy/XHNTs nanocomposites.

The results of DMTA measurements containing storage modulus and damping factor (tanδ) are demonstrated in Figure 8. It is clear that the incorporation of XNBR grafted nanotubes cause a rise in the storage modulus of XNBR/epoxy nanocomposites due to the stiffening effect of XHNTs [47] along with their interactions with the polymer matrix. Figure 8a revealed that the effect of nanotubes on storage modulus of nanotubes could enhanced up to 42% with the addition of 7 wt % of XHNTs to the XNBR/epoxy matrix. Nevertheless, as depicted in Figure 8b there is a decrease in the results of tanδ with higher concentrations of XHNTs signifying a more elastic behavior of prepared nanocomposites due to the induction of some restrictions in chain mobility of polymer matrix by the incorporation of XNBR grafted nanotubes [48].

### 4.6. Swelling Characteristics

**Figure 9:**
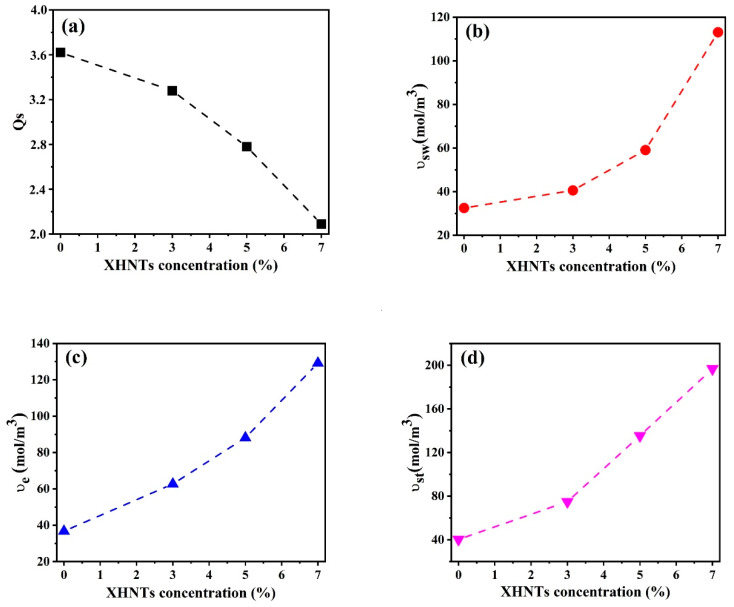
Swelling behavior of XNBR/epoxy nanocomposites containing various XHNTs concentrations (**a**) swelling ratio, (**b**) cross-link density resulted from swelling analysis, (**c**) cross-link density resulted from stress–strain measurements, (**d**) cross-link density resulted from dynamic mechanical thermal analysis (DMTA).

The results of calculated swelling ratio and cross-link density for various XNBR/epoxy/XHNTs nanocomposites as a function of XHNTs loading were demonstrated in Figure 9. It is obvious from Figure 9a that the higher XHNTs concentrations leads to a decrease of swelling ratio more than 40% in comparison with the neat XNBR/epoxy compound. Conversely, the results of cross-link density presented in Figure 9b revealed that the higher XHNTs content in the nanocomposites increases this parameter which provides more interlocking effect of nanotubes due to its physical structure and some interactions with the polymer matrix [49]. Therefore, the extent of solvent diffusion and swelling in the bulk of nanocomposites was limited by the introduction of nanotubes into the XNBR/epoxy matrix.

The cross-link density calculated in accordance with the stress–strain curves and storage modulus were investigated in Figure 9c,d, respectively. It is obvious that the results of cross-link density obtained from stress–strain behavior and storage modulus data are greater than the cross-link density resulted from the swelling measurements. It may be due that the swelling measurements were carried out at an equilibrium state [50], whereas the stress–strain and storage modulus were obtained under instabilities resulted from the elongations of samples [51]. Furthermore, the swelling behavior of XNBR/epoxy/XHNTs nanocomposites with XHNTs concentration show different trends in various approaches as indicated in Figure 9b–d. As revealed in Figure 9b, the cross-link density of rubber nanocomposites increases with higher rates from 5 wt % to 7 wt % XHNTs. However, this increment was observed from 3 wt % to 7 wt % XHNTs in cross-link density resulted from stress–strain and storage modulus approaches as indicated in Figure 9c,d.

## 5. Conclusions

The XNBR/epoxy nanocomposites containing various concentrations of XNBR grafted halloysite nanotubes (XHNTs) were prepared through using a two roll mills. The results of cure rheometer show that the introduction of XHNTs into the XNBR/epoxy matrix cause a rise in the maximum torque while decreases the scorch and optimum cure times. The morphology investigations of nanocomposites revealed a rougher fracture surface of nanocomposites with a unique dispersion of nanotubes into the rubber matrix. Tensile strength and modulus at 300% elongations increased with higher nanotubes loadings while there are some decreases in the values of elongation at break. The theoretical evaluations for uniaxial stress–strain behavior of XNBR/epoxy/XHNTs nanocomposites cleared that some of material parameters of used Bergström–Boyce model significantly varied with the XHNTs concentrations. Nevertheless, the model could precisely predict the large strain hyperelastic behavior of XNBR/epoxy/XHNTs nanocomposites. However, there is some deviations from experimental values of stress–strain data at the regions of ultimate elongations. Furthermore, the results of theoretical investigations show a more deviation from experimental values with higher loadings of XNBR grafted nanotubes. The results of dynamic mechanical analysis demonstrated a higher values of storage modulus with higher loadings of XHNTs, while there is some diminution of damping factor. Swelling studies of nanocomposites suggested that the nanotubes act as obstacle to reduce the diffusion of the used solvent into the bulk of rubber matrix. The results preferred to make a rubber based nanocomposite with higher mechanical behavior which could be prepared through using general mixing equipment.

## Figures and Tables

**Table 1:** Formulations of various carboxylated nitrile butadiene rubber (XNBR)/epoxy/ XNBR grafted halloysite nanotube (XHNT) nanocomposites (in parts per hundred rubber (phr)).

Sample Code	XNBR/Epoxy (70/30)	XHNT	ZnO	Stearic Acid
XE15	100	0	6	2
XE15H3	100	3	6	2
XE15H5	100	5	6	2
XE15H7	100	7	6	2

**Table 2:** Cure characteristics of various XNBR/epoxy/XHNTs nanocomposites.

Sample Code	*Mh* (lb_f_.in) ^a^	*Ml* (lb_f_.in) ^b^	(*Mh*-*Ml*) (lb_f_.in)	*ts*2 (min) ^c^	*tc*90 (min) ^d^
XE15	66.60	18.77	47.83	3.39	20.3
XE15H3	66.80	19.73	47.07	2.75	19.9
XE15H5	67.60	21.69	45.91	2.68	19.5
XE15H7	69.90	24.07	45.83	2.51	18.8

^a^ Maximum torque value; ^b^ Minimum torque value; ^c^ Scorch time; ^d^ Optimum cure time.

**Table 3:** Material model parameters for various XNBR/epoxy/XHNTs nanocomposites.

Sample Codes	μ (MPa)	κ (MPa)	*S*	ζ	*C*	τ_base_	m	τ_cut_
**XE15**	0.23	29.67	103.14	3.62 × 10^−9^	−0.89	0.39	4.06	0.001
**XE15H3**	0.24	39.31	103.15	1.58 × 10^−7^	−0.78	0.44	4.14	0.033
**XE15H5**	0.26	47.79	103.17	1.96 × 10^−7^	−0.76	0.48	4.15	0.060
**XE15H7**	0.29	55.40	103.18	1.97 × 10^−7^	−0.65	0.49	4.21	0.070
